# Brain and mental: two sides of the same coin

**DOI:** 10.1192/j.eurpsy.2025.77

**Published:** 2025-08-26

**Authors:** P. Falkai

**Affiliations:** Psychiatry, Munich University Hospital, Munich, Germany

## Abstract

**Abstract:**

**Mental health is an integral and essential component of health.** An important implication of this definition is that mental health is more than just the absence of mental disorders or disabilities. **Brain health can be defined as a life-long dynamic state of cognitive, emotional and motor domains underpinned by physiological processes. Exercise is a good model, since training the body improves Brain and Mental Health. Pathophysiological research and biomarkers are required.**

**Disclosure of Interest:**

None Declared

